# Umbilical Cord Blood pH Level, Apgar Score, and Attention-Deficit/Hyperactivity Disorder

**DOI:** 10.1001/jamanetworkopen.2025.54672

**Published:** 2026-01-26

**Authors:** Mette Vestergård Pedersen, Morten Søndergaard Lindhard, Dag Moster, Rolv Terje Lie, Tine Brink Henriksen

**Affiliations:** 1 Department of Pediatrics and Adolescent Medicine, Aarhus University Hospital, Aarhus, Denmark; 2 Department of Clinical Medicine, Aarhus University, Aarhus, Denmark; 3 Department of Pediatrics, Randers Regional Hospital, Randers, Denmark; 4 Department of Global Public Health and Primary Care, University of Bergen, Bergen, Norway; 5 Department of Neonatal Intensive Care, Haukeland University Hospital, Bergen, Norway

## Abstract

**Importance:**

Perinatal hypoxia is an important cause of neurodevelopmental impairment and may thus be associated with attention-deficit/hyperactivity disorder (ADHD). In population-based studies, hypoxia is often assessed by Apgar score alone; however, criteria for relevant perinatal hypoxia require both clinical and biochemical abnormalities (eg, a low Apgar score and a low umbilical cord blood pH level).

**Objectives:**

To identify full-term and near-term newborns exposed to hypoxia by combining the Apgar score and the umbilical cord blood pH level and to investigate their association with ADHD.

**Design, Setting, and Participants:**

This cohort study, with follow-up from birth until December 31, 2022, identified all singleton newborns with a gestational age of 35 weeks or more, without major malformations, born between January 1, 2004, and December 31, 2018, using a Danish nationwide registry. Data were analyzed from October 2024 to November 2025.

**Exposure:**

Combinations of a 5-minute Apgar score category (0-3, 4-6, and 7-10) and an umbilical cord blood pH category (<7.10, 7.10-7.19, and ≥7.20). Newborns with an Apgar score of 7 to 10 combined with a pH level of 7.20 or higher were considered the reference group.

**Main Outcomes and Measures:**

The primary outcome was ADHD identified by hospital contacts with ADHD diagnosis and/or prescriptions of ADHD medication. Outcome measures were odds ratios adjusted for relevant confounders. Multiple imputation was used to handle missing data.

**Results:**

Of 819 658 newborns, 419 581 (51.2%) were male, and 429 492 (52.5%) were born 39 to 40 weeks of gestational age. Both a low Apgar score of 0 to 3 and a pH less than 7.10 were observed for 249 newborns (0.3%). Increased odds of ADHD was observed for most combinations of a pH of less than 7.20 combined with an Apgar score of less than 7 compared with the reference group and was most pronounced when the Apgar score was 0 to 3 and the pH was less than 7.10 (adjusted odds ratio, 1.86 [95% CI, 1.04-3.33]). If the pH was higher than 7.20, there was no association between Apgar score and ADHD. Similarly, if the Apgar score was 7 or higher, there was no association between pH and ADHD.

**Conclusions and Relevance:**

In this cohort study, an increased odds of ADHD was observed when clinical and biochemical measures of hypoxia were combined as expressed by a low Apgar score and a low umbilical cord pH level at birth. However, when either the Apgar score or the umbilical cord blood pH level was normal, perinatal hypoxia seemed less likely, and no increased odds of ADHD was observed.

## Key Points

**Question:**

Is perinatal hypoxia assessed by clinical and biochemical measures associated with attention-deficit/hyperactivity disorder (ADHD)?

**Findings:**

In this nationwide cohort study of 819 658 singleton newborns, with a gestational age of 35 weeks or more, the combination of an Apgar score of less than 7 and an umbilical cord blood pH level of less than 7.20 were associated with ADHD. When the Apgar score was normal, there was no association between pH level and ADHD; when the pH level was normal, there was no association between Apgar score and ADHD.

**Meaning:**

These findings suggest that perinatal hypoxia was associated with an increased risk of ADHD only when both clinical and biochemical features were affected.

## Teaser

This cohort study evaluates whether perinatal hypoxia, defined as low Apgar score and low umbilical cord blood pH level), is associated with subsequent attention-deficit/hyperactivity disorder (ADHD) diagnosis among Danish near-term and full-term infants.

## Introduction

Perinatal hypoxia is an important cause of mortality and long-term disability.^1^ Impaired peripartum gas exchange leading to hypoxia and ischemia can affect cellular metabolism and cause neuronal cell death, which may lead to brain damage and neurodevelopmental impairment.^2^ Attention-deficit/hyperactivity disorder (ADHD) is a neurodevelopmental disorder characterized by inappropriate levels of inattention, hyperactivity, and impulsivity.^3^ Its prevalence is increasing, with the latest studies reporting up to 8% of children and adolescents worldwide living with ADHD.^4^ Perinatal hypoxia may also affect behavioral modalities and thus may play a role in the development of ADHD.^5^

To identify newborns exposed to hypoxia, both clinical and biochemical measures should be affected (eg, the Apgar score and umbilical cord blood pH level).^6^ The Apgar score was developed as a clinical tool to evaluate the physical condition of infants shortly after birth.^7^ However, a low Apgar score is unspecific as numerous causes, including hypoxia, may lead to a low score. Other causes are perinatal infection and inflammation, congenital neurological disease, chronic placental insufficiency, maternal medication, and more.^8,9^ Despite the consistency in the findings from studies that report increased risk of ADHD for newborns with low Apgar scores, the nature of this measure is not qualified, even though studies have used a low Apgar score as the sole measure of hypoxia without validating this putative cause.^5,10,11^ Umbilical arterial blood gas analyses, including pH level, are often used as a biochemical measure to determine whether peripartum gas exchange was impaired and the newborn therefore exposed to hypoxia.^6,8^ As for Apgar score and umbilical pH alone at various levels, they have been associated with long-term neurodevelopmental impairment, including ADHD.^12,13^ However, a low umbilical cord pH level may well be present without the clinical features of hypoxia.^6^

The association between perinatal hypoxia assessed by combining clinical and biochemical features and ADHD remains poorly examined.^5,13^ In this study, we aim to identify full-term and near-term newborns exposed to relevant hypoxia by combining Apgar score and umbilical cord blood pH and to investigate the association with ADHD.

## Methods

This cohort study was approved by Central Denmark Region, and data access was granted by the Danish Health Data Authorities. Informed consent was waived because the study was based on deidentified registry data. The study followed the Strengthening the Reporting of Observational Studies in Epidemiology (STROBE) reporting guideline.^14^

### Study Design and Setting

This population-based cohort study was based on data from nationwide registries in Denmark. All inhabitants of Denmark are assigned a unique 10-digit civil registration number, which allows data linkage across registries at an individual level.^15^

### Study Population

**Figure 1.**
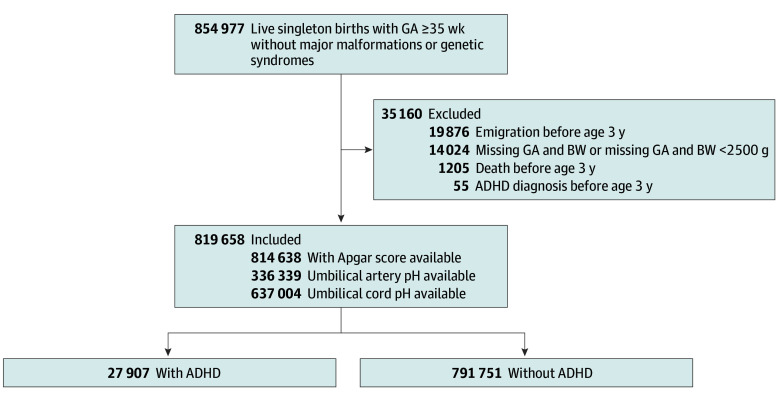
Study Cohort Flowchart, Denmark 2004-2018 ADHD indicates attention-deficit/hyperactivity disorder; BW, birth weight; and GA, gestational age.

We included all live-born, singleton newborns with a gestational age of 35 weeks or more, born between January 1, 2004, and December 31, 2018. Newborns with missing gestational age and birth weight or missing gestational age and birth weight less than 2500 g were excluded. We also excluded newborns with chromosomal abnormalities or any major malformation of the heart, respiratory system, nervous system, or gastrointestinal tract according to a Danish modification of the EUROCAT (European network of population-based registries for the epidemiological surveillance of congenital anomalies) classification.^16^ The children were followed up until December 31, 2022. Children who died, emigrated, or received a diagnosis of ADHD before 3 years of age (without confirmation after 3 years of age) were excluded (Figure 1).

### Exposure

Apgar scores at 5 minutes and umbilical cord blood pH levels were retrieved from the Danish Medical Birth Registry. The Apgar score at 5 minutes was categorized into 3 groups (0-3, 4-6, and 7-10), according to previous studies and international conventions.^8^ When 2 pH values were registered and the difference between these was 0.02 or more, they were considered from the artery and vein.^17^ Otherwise, both samples were considered venous. If only 1 pH value was registered, it was considered from the vein.^18^ We used the lowest registered pH for the main analysis, regardless of this being from the artery or vein, and refer to this as pH unless otherwise stated. pH values less than 6.50 and more than 7.70 were considered invalid and recoded as missing.^19^ We categorized pH levels into 3 groups according to previous studies (<7.10, 7.10-7.19, and ≥7.20).^12,20^ Newborns with an Apgar score of 7 to 10 and a pH of 7.20 or higher were used as the reference category.^18^

### Outcome

To identify children with ADHD, we used diagnoses from inpatient and outpatient contacts in the Danish National Patient Registry, the Danish Psychiatric Central Registry, and prescriptions from the Danish National Prescription Registry. ADHD was identified as a hyperkinetic or attention-deficit diagnosis (codes F90.0, F90.1, F90.8, F90.9, and F98.8) according to the *International Statistical Classification of Diseases and Related Health Problems, Tenth Revision* (*ICD-10*). Prescriptions assumed to treat ADHD included dexamphetamine (code N06BA02), methylphenidate (code N0BA04), modafinil (code N06BA07), atomoxetin (code N06BA09), and lisdexamphetamine (code N06BA12) according to the Anatomical Therapeutic Chemical classification system. To minimize misclassification, we defined ADHD as (1) at least 2 contacts with an *ICD-10* diagnosis, (2) 1 contact with an *ICD-10* diagnosis combined with at least 1 prescription, or (3) at least 2 prescriptions.

### Covariates

Relevant covariates were identified from the literature and depicted by use of directed acyclic graphs (eFigure in Supplement 1). Data sources are presented in eTable 1 in Supplement 1.

### Statistical Analysis

The association of the combination of Apgar score and pH with ADHD was estimated using multivariable logistic regression. In the crude model, we adjusted for birth year. To consider changes in diagnostic criteria and prevalence of ADHD over time, birth year was modeled as a categorical variable using 1-year intervals. In the adjusted model, we additionally adjusted for sex, gestational age, birth weight, maternal age, parity, smoking in pregnancy, maternal history of psychiatric disease, maternal education, household income, and the parents’ country of origin. Covariates were included in the adjusted model, categorized as shown in Table 1. In all analyses, nonindependence between siblings was accounted for by clustering observations with the same mother, providing robust standard errors for the 95% CIs. We used multiple imputation by chained equations for missing values (eMethods in Supplement 1).^21^ By categorizing the exposure according to both the Apgar score and the pH level, a potential interaction between them was embedded in the model. The interaction was then evaluated by investigating whether the log-linear trend of Apgar score categories was different between pH categories by use of the Wald test.

**Table 1.** Characteristics of All Singleton Live Births With Gestational Age of 35 or More Weeks in Denmark, 2004-2018

Characteristic	Newborns, No. (%)^a^				
	Total population	Apgar score <7^b^	pH <7.10^c^	Apgar score <7 and pH <7.10^d^
Total	819 658 (100)	4446 (0.5)	22 813 (2.8)	1090 (0.1)
Sex				
Female	400 077 (48.8)	1888 (42.5)	10 381 (45.5)	486 (44.6)
Male	419 581 (51.2)	2558 (57.5)	12 432 (54.5)	604 (55.4)
Birth year				
2004-2008	286 485 (35.0)	1417 (31.9)	5306 (23.3)	280 (25.7)
2009-2013	265 225 (32.3)	1477 (33.2)	7183(31.5)	350 (32.1)
2014-2018	267 948 (32.7)	1552 (34.9)	10 324 (45.2)	460 (42.2)
Gestational age, wk				
35-36	23 626 (2.9)	286 (6.5)	457 (2.0)	54 (5.0)
37-38	151 955 (18.6)	822 (18.6)	2854 (12.5)	132 (12.2)
39-40	429 492 (52.5)	1989 (45.0)	11 365 (49.9)	510 (47.1)
≥41	212 435 (26.0)	1321 (29.9)	8085 (35.5)	386 (35.7)
Missing	2150 (0.3)	28 (0.6)	52 (0.2)	8 (0.7)
Birth weight, g				
<3000g	101 314 (12.4)	756 (17.6)	2656 (11.7)	151 (14.7)
3000-4000	565 741 (69.3)	2653 (61.7)	15 444 (68.1)	652 (63.4)
>4000	149 112 (18.2)	889 (20.7)	4564 (20.2)	225 (21.9)
Missing	3491 (0.4)	148 (3.3)	149 (0.7)	62 (5.6)
Parity				
First born	376 379 (45.9)	2626 (59.1)	14 562 (63.8)	652 (59.8)
Second born or higher	443 279 (54.1)	1820 (40.9)	8251 (36.2)	438 (40.2)
Maternal age, y				
<20	10 284 (1.3)	63 (1.4)	286 (1.3)	16 (1.5)
20-35	686 681 (83.8)	3659 (82.2)	19 212 (84.2)	893 (81.9)
>35	122 693 (15.0)	724 (16.3)	3315 (14.5)	181 (16.6)
Maternal history of psychiatric disease	75 794 (9.3)	523 (11.8)	2240 (9.8)	120 (11.0)
Smoking in pregnancy	102 492 (12.8)	622 (14.4)	2492 (11.2)	139 (13.2)
Missing	16 995 (2.1)	134 (3.0)	489 (2.1)	38 (3.5)
Maternal education				
No education or lower secondary	131 961 (16.3)	818 (18.7)	3033 (13.4)	192 (17.8)
Upper secondary	327 578 (40.4)	1838 (41.9)	8986 (39.7)	428 (39.7)
Higher education	350 644 (43.3)	1727 (39.4)	10 614 (46.9)	459 (42.5)
Missing	9475 (1.2)	63 (1.4)	180 (0.8)	11 (1.0)
Family income quartile				
First quartile	204 914 (25.0)	1104 (24.9)	4800 (21.0)	258 (23.7)
Second quartile	204 914 (25.0)	1103 (24.8)	4972 (21.8)	260 (23.9)
Third quartile	204 914 (25.0)	1112 (25.0)	6081 (26.7)	284 (26.0)
Fourth quartile	204 915 (25.0)	1127 (25.3)	6960 (30.5)	288 (26.4)
Parents’ country of origin				
At least 1 parent is of Western origin^e^	757 588 (92.4)	4132 (92.9)	21 492 (94.2)	1015 (93.1)

^a^ All percentages are calculated from valid (nonmissing) information.

^b^ Apgar score was missing among 5020 of the 819 658 newborns in the total population (0.6%). Percentages are of all newborns with available Apgar score.

^c^ Umbilical cord blood pH was missing among 180 907 of the 819 658 newborns in the total population (22.0%). Percentages are of all newborns with available umbilical cord blood pH.

^d^ Apgar score or umbilical cord blood pH was missing among 182 654 of the 819 658 newborns in the total population (22.3%). Percentages are of all newborns with available Apgar score and available umbilical cord blood pH.

^e^ Birthplace or citizenship in the European Union, Great Britain, Iceland, Liechtenstein, Monaco, Switzerland, Norway, Canada, United States, Australia, or New Zealand.

To evaluate the main analysis, we conducted sensitivity analyses using only observations with complete data and an analysis using only newborns with a verified umbilical arterial pH value. The multiple imputation model was further evaluated by reporting the distributions of observed and imputed Apgar scores and pH values and by randomly removing observed Apgar scores and observed pH values, imputing these, and reporting the distributions.

ADHD is a chronic condition likely to be present also before the time of diagnosis. Therefore, we chose logistic regression as the primary analysis. As the outcome was rare, the odds ratios (ORs) were interpreted as relative risks.^22^ We conducted a log binomial regression and a Cox proportional hazards regression to estimate relative risk and hazard ratios, respectively.

Registration of umbilical cord blood pH in the Danish Medical Birth Register started in 2004 and became a part of the national quality improvement initiative in 2012.^23^ In a sensitivity analysis with aggregated exposure categories, we stratified by birth years 2004 to 2011 and 2012 to 2018. Other severe neurologic impairments may interfere with the diagnosis of ADHD. We conducted an analysis that excluded newborns with cerebral palsy, intellectual disability, and epilepsy. To control for shared unmeasured confounding, we performed a sibling analysis (eAppendix in Supplement 1). A 2-sided *P* < .05 was considered statistically significant. All statistical analysis was performed using Stata Statistical Software, version 18 (StataCorp, LLC). Data were analyzed from October 2024 to November 2025.

## Results

### Population Characteristics

The study population included 819 658 newborns, of whom 419 581 (51.2%) were male and 429 492 (52.5%) were born 39 to 40 weeks of gestational age (Table 1). A low Apgar score of 0 to 3 combined with a pH of less than 7.10 was observed for 249 newborns (0.3%) (Table 2). Of 22 813 newborns with a pH of less than 7.10, 10 324 (45.2%) were born between 2014 and 2018.

**Table 2.** Prevalence of ADHD and Median Age at Diagnosis by Exposure Categories Among Children Born at 35 or More Weeks’ Gestation in Denmark, 2004-2018

Apgar score per pH category	Total No. (N = 819 658)	ADHD, No. (%) (n = 27 907)	Age at diagnosis, median (IQR), y^a^
pH ≥7.20			
7-10	496 395	15 170 (3.1)	9.3 (7.4-11.9)
4-6	1199	38 (3.2)	9.1 (7.3-10.9)
0-3	383	13 (3.4)	8.6 (7.3-10.4)
pH 7.10-7.19			
7-10	115 505	2970 (2.6)	8.9 (7.0-11.2)
4-6	724	30 (4.1)	8.9 (7.6-10.5)
0-3	187	8 (4.3)	9.4 (8.8-10.8)
pH <7.10			
7-10	21 521	620 (2.9)	9.1 (7.0-11.9)
4-6	841	30 (3.6)	10.4 (6.7-14.5)
0-3	249	12 (4.8)	7.5 (5.1-10.7)
Either is missing	182 654	9016 (4.9)	10.8 (8.3-14.2)

Abbreviation: ADHD, attention-deficit/hyperactivity disorder.

^a^ Due to Danish data protection legislation, the median (IQR) values are based on pseudopercentiles averaging 5 values around each position.

Umbilical cord blood pH values were missing for 180 907 of the 819 658 newborns (22.1%), with higher proportions of missing pH values in the oldest birth cohorts. Apgar scores were missing for 5020 of 819 658 newborns (0.6%). Missing data on covariates are presented in Table 1.

In total, 27 907 of 819 658 newborns (3.4%) developed ADHD (Figure 1). Of 249 newborns with a pH of less than 7.10 and an Apgar score of 0 to 3, ADHD occurred among 12 (4.8%) newborns. The occurrence of ADHD was 15 170 among the 496 395 newborns (3.1%) within the reference category (Table 2). The median age at diagnosis in the reference category was 9.3 years (IQR, 7.4-11.9). For children with a pH of less than 7.10 and an Apgar score of 0 to 3, the median age at diagnosis was 7.5 years (IQR, 5.1-10.7) (Table 2).

### Main Results

**Figure 2.**
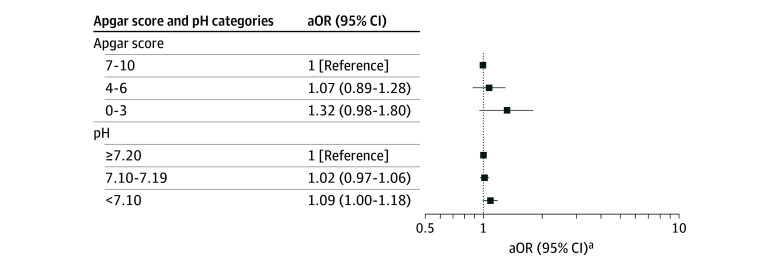
Associations Between 5-Minute Apgar Score Alone and Attention-Deficit/Hyperactivity Disorder (ADHD) and Between Umbilical Cord Blood pH Alone and ADHD in Children Born at 35 or More Weeks’ Gestation in Denmark, 2004-2018 aOR indicates adjusted odds ratio. ^a^Logistic regression using imputed data adjusted for birth year, sex, gestational age, birth weight, maternal age, parity, smoking in pregnancy, maternal history of psychiatric disease, maternal education, family income, and child ethnicity.

**Figure 3.**
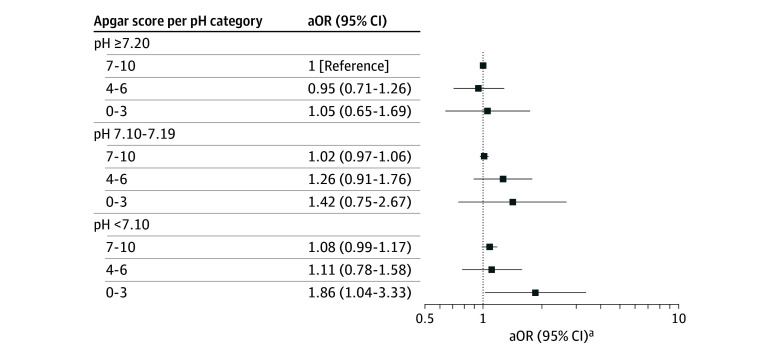
Association Between Umbilical Cord Blood pH Combined With 5-Minute Apgar Score and Attention-Deficit/Hyperactivity Disorder (ADHD) in Children Born at 35 or More Weeks’ Gestation in Denmark, 2004-2018 aOR indicates adjusted odds ratio. ^a^Logistic regression using imputed data adjusted for birth year, sex, gestational age, birth weight, maternal age, parity, smoking in pregnancy, maternal history of psychiatric disease, maternal education, family income, and child ethnicity. Log-linear trend of Apgar between pH categories using the Wald test (*P* = .32).

The associations of Apgar score alone with ADHD and of pH alone with ADHD are shown in Figure 2. Estimates are presented in eTables 2 and 3 in Supplement 1. When Apgar score and pH were combined, the point estimates suggested increased odds of ADHD for most categories with pH less than 7.20 combined with an Apgar score of less than 7 compared with the reference group. The highest OR was observed for newborns with the lowest Apgar score and the lowest pH (adjusted OR, 1.86 [95% CI, 1.04-3.33]). In the categories with reduced pH, the OR increased with decreasing Apgar score. By visual inspection, this pattern was not seen if the pH was higher than 7.20, but the Apgar score trend was not statistically different between pH categories (Figure 3).

For all newborns with pH levels higher than 7.20, there was no association between Apgar score and ADHD. Likewise, if the Apgar score was in the category of 7 to 10, there was no association between pH and ADHD (Figure 3). All estimates are presented in eTable 4 in Supplement 1.

ORs of ADHD in aggregated exposure groups were comparable for children born between 2004 and 2011 and children born between 2012 and 2018 (eTables 10 and 11 in Supplement 1). In all sensitivity analyses, the point estimates were comparable to those of the main analysis (eTables 5-9 in Supplement 1). The result of the sibling analysis is presented in the eAppendix in Supplement 1, and the evaluation of the imputation model is presented in eTables 12 and 13 in Supplement 1.

## Discussion

In this population-based cohort study, we identified newborns exposed to relevant perinatal hypoxia by combining the 5-minute Apgar score and the umbilical cord blood pH level, and we observed an association between the combination of these 2 measures with increased risk of ADHD. If the Apgar score was low and the pH level was normal, or vice versa, we observed no association with ADHD.

Consensus on a definition for the term *asphyxia* to describe a newborn supposed to be exposed to peripartum hypoxia is lacking, and we therefore chose not to use the term *asphyxia* in this study.^8,24,25^ The American Academy of Pediatrics and the American College of Obstetricians and Gynecologists address the need for considering both clinical and biochemical measurements when assuming that peripartum hypoxia and ischemia have been present.^6,8^ A meta-analysis found that perinatal hypoxia was associated with ADHD but used the Apgar score alone or other exposures in which compromised gas exchange may be more frequent but not always present, such as umbilical cord prolapse and breech or transverse presentation.^5^ We also observed increased odds of ADHD for newborns with a low Apgar score if pH was not considered. A low Apgar score from other causes than perinatal hypoxia, such as disease that may exist before birth or maternal drug exposure, may be benign if handled appropriately.^8^ Reporting increased risk of ADHD from such benign causes may lead to unnecessary concern in parents and health care personnel. The 95% CIs were wide, but the point estimates suggested no increased risk of ADHD if the Apgar score was low but the pH was normal. Likewise, if only the pH was low but the Apgar score was normal, which was the case for most children within the low pH category, the risk of ADHD was not increased. Thus, our findings suggest that these children should not be followed up for behavioral symptoms based on only their Apgar score or their umbilical cord blood pH. The large proportion of newborns with data on umbilical cord blood pH in this population-based study allowed us to follow the American Academy of Pediatrics and American College of Obstetricians and Gynecologists statements and assess perinatal hypoxia by combining Apgar score and umbilical cord blood pH.^6,8^ The observed point estimates suggested that the risk of ADHD was only increased when both measures were low (ie, pH values <7.20 in combination with 5-minute Apgar scores <7). Despite the increased OR, the absolute risk of ADHD remained low among children with both a low Apgar score and a low pH, suggesting that perinatal hypoxia may be only 1 of several causes of ADHD in full-term and near-term newborns.

To the best of our knowledge, the only previous study on the combination of Apgar score and umbilical cord blood pH for diagnosing ADHD was a Finnish cohort study of 295 687 newborns.^13^ It included infants who were also born preterm, used 1-minute Apgar score, and categorized umbilical cord blood pH into 2 groups (ie, <7.15 and ≥7.15). The study reported some 50% increased risk of ADHD for newborns with an Apgar score of 0 to 3 at 1 minute in both pH groups. A 1-minute Apgar score is not available in the national Danish registers, and therefore this information was unavailable for our analyses. Most newborns with a low 1-minute Apgar score will, with sufficient support, achieve higher and even normal Apgar scores at 5 minutes.^8,26,27,28^ According to our results, these infants will have no increased risk of ADHD. By using the 5-minute Apgar score only, more pronounced and possibly also prolonged hypoxic events are included. The Finnish cohort included approximately 40% of the population.^23^ We included an entire population of full-term and near-term infants consisting of almost 3 times as many newborns as the Finnish cohort, enabling us to make several clinical and biochemical categories depicting different severities of perinatal hypoxia. Furthermore, we were able to assess the robustness of our results by rigorous sensitivity analyses.

### Limitations

This study has some limitations. Umbilical cord blood pH registrations were incomplete in the first years after 2004. In the first years, only 1 value was registered, making us unable to validate whether it was of arterial or venous origin. The acidosis may have been worse than the venous registrations indicate.^17,18^ This would underestimate the ORs in the lowest pH categories. We observed comparable point estimates when using only validated umbilical artery pH measures in most exposure groups; however, power was limited in this analysis.

A pH cutoff of 7.00 in umbilical cord blood is commonly used, together with clinical symptoms, to identify infants at high risk of death or brain injury.^6^ We used a pH cutoff of 7.10. Ideally, the pH categories should have been more granulated below 7.10, but the numbers were too low for meaningful interpretations. When not considering Apgar score, the risk of ADHD among children with a pH of less than 7.00 did not differ from that of those with a pH of 7.00 to 7.09.

Missing pH may be associated with the course of birth and the condition of the newborn.^23^ Due to our extensive information about this, we assumed data to be missing at random as the probability of data being missing was assumed to depend on the observed data and not the unobserved data.^21^ When including several auxiliary variables in the imputation model, the point estimates may be reduced.^29^ Point estimates from our analysis using imputed data were comparable but slightly reduced compared with estimates from the analysis using only complete data. In all Apgar score categories, lower proportions of children were imputed with a pH of less than 7.10 than of children with this pH observed, which could mean that missing pH was more common among noncomplicated births or that the imputation model was less likely to impute extreme values. When randomly removing observed Apgar scores or observed pH levels and afterward imputing them, the observed and imputed distributions within categories were comparable.

The median age at ADHD diagnosis was slightly lower for the children with the lowest pH levels and lowest Apgar scores compared with the reference group. Children with perinatal hypoxia may have easier hospital access, but their parents may also be more likely to seek medical advice for their children. For these reasons, children with perinatal hypoxia may be more likely to receive an ADHD diagnosis, and this may be given earlier than for other children. This would tend to amplify the risk of ADHD in children with perinatal hypoxia when using shorter follow-up periods.^30^ Yet, an early diagnosis could also be due to more severe symtoms.^31^ We were able to follow up with the children born in 2018 until 4 years of age. Danish guidelines state that ADHD can be diagnosed as early as 3 years of age.^32^ However, diagnosis is more common at 8 to 10 years of age.^31^ The oldest birth cohorts had higher proportions of missing pH but also a higher prevalence of ADHD due to longer follow-up. When dividing the cohort of children born between 2004 and 2011 and children born between 2012 and 2018, the ORs of ADHD in the aggregated exposure group were comparable between these 2 cohorts, also when applying the same follow-up time to both cohorts. Patterns from the Cox proportional hazards regression were similar to those observed in the logistic regression, where time at risk was disregarded.

Severe perinatal hypoxia with low Apgar score and pH may cause other neurologic disabilities, such as cerebral palsy, intellectual disability, and epilepsy.^12,33,34^ These conditions may co-occur with and lead to more frequent diagnoses of ADHD. When excluding children with cerebral palsy, intellectual disability, or epilepsy, we still observed an increased risk of ADHD for newborns exposed to hypoxia without other severe neurologic disabilities.

In 2007 and 2008, therapeutic hypothermia was introduced for moderate to severe hypoxic ischemic encephalopathy in Denmark. The treatment reduces the risk of cerebral palsy and death, but the effect on behavioral deficits such as ADHD is less well investigated.^35^ The basal ganglia are known to be sensitive to hypoxia and are also thought to be affected in ADHD.^36,37,38^ Therapeutic hypothermia reduces structural abnormalities in the basal ganglia after perinatal hypoxia and could therefore reduce the risk of ADHD.^39^ We adjusted for year of birth and thereby considered overall improvements in neonatal care over time, including therapeutic hypothermia.

## Conclusions

In this cohort study, children with evidence of relevant perinatal hypoxia assessed by the combination of a low Apgar score and a low umbilical cord blood pH had a higher risk of ADHD than children without hypoxia at birth. When either the Apgar score or the pH was normal, we found no increased risk of ADHD compared with children who had both a normal Apgar score and a normal pH level. These findings are important as they may reduce anxiety in the parents of these large groups of children.

## Notes

Supplement 1. **eFigure.** Directed Acyclic Graph on the association between perinatal hypoxia and ADHD **eTable 1.** Data sources **eMethods.** Multiple imputation model **eAppendix.** Sibling analysis **eTable 2. **The association between Apgar score alone or umbilical cord blood pH alone and ADHD **eTable 3.** The association between umbilical cord blood pH alone and ADHD. Including a separate category for pH<7.00 **eTable 4.** The association between combinations of Apgar score and umbilical cord blood pH and ADHD using imputed data **eTable 5.** Complete data analysis **eTable 6.** Analysis with exposure categorized according to validated arterial values **eTable 7.** Log binomial regression analysis **eTable 8.** Cox proportional hazards regression analysis **eTable 9.** Analysis excluding children with cerebral palsy, intellectual disability, and epilepsy **eTable 10.** The association between combinations of Apgar score and umbilical cord blood pH and ADHD in children born 2004-2011 and children born 2012-2018 using complete data **eTable 11.** The association between combinations of Apgar score and umbilical cord blood pH and ADHD in children born 2004-2011 and children born 2012-2018 using imputed data **eTable 12.** Distribution of observed and imputed Apgar score **eTable 13.** Distribution of observed and imputed umbilical cord pH according to Apgar score category
eReferences

Supplement 2.
Data Sharing Statement
