# Supplementary material for: Umbilical Cord Blood pH Level, Apgar Score, and Attention-Deficit/Hyperactivity Disorder
Source: JAMA Netw Open. 2026 Jan 26;9(1):e2554672. doi: 10.1001/jamanetworkopen.2025.54672 (PMC12836134; doi:10.1001/jamanetworkopen.2025.54672)
Supplement: Supplement 1. — eFigure. Directed Acyclic Graph on the association between perinatal hypoxia and ADHD eTable 1. Data sources eMethods. Multiple imputation model eAppendix. Sibling analysis eTable 2. The association between Apgar score alone or umbilical cord blood pH alone and ADHD eTable 3. The association between umbilical cord blood pH alone and ADHD. Including a separate category for pH<7.00 eTable 4. The association between combinations of Apgar score and umbilical cord blood pH and ADHD using imputed data eTable 5. Complete data analysis eTable 6. Analysis with exposure categorized according to validated arterial values eTable 7. Log binomial regression analysis eTable 8. Cox proportional hazards regression analysis eTable 9. Analysis excluding children with cerebral palsy, intellectual disability, and epilepsy eTable 10. The association between combinations of Apgar score and umbilical cord blood pH and ADHD in children born 2004-2011 and children born 2012-2018 using complete data eTable 11. The association between combinations of Apgar score and umbilical cord blood pH and ADHD in children born 2004-2011 and children born 2012-2018 using imputed data eTable 12. Distribution of observed and imputed Apgar score eTable 13. Distribution of observed and imputed umbilical cord pH according to Apgar score category eReferences [file jamanetwopen-e2554672-s001.pdf]

Supplemental Online Content

Pedersen M, Lindhard MS, Moster D, Lie RT, Henriksen TB. Umbilical cord pH level, Apgar score, and attention-deficit/hyperactivity disorder. *JAMA Netw Open*. 2026;9(1):e2554672. 10.1001/jamanetworkopen.2025.54672

eFigure. Directed Acyclic Graph on the association between perinatal hypoxia and ADHD
eTable 1. Data sources
eMethods. Multiple imputation model
eAppendix. Sibling analysis
eTable 2. The association between Apgar score alone or umbilical cord blood pH alone and ADHD
eTable 3. The association between umbilical cord blood pH alone and ADHD. Including a separate category for pH<7.00
eTable 4. The association between combinations of Apgar score and umbilical cord blood pH and ADHD using imputed data
eTable 5. Complete data analysis
eTable 6. Analysis with exposure categorized according to validated arterial values
eTable 7. Log binomial regression analysis
eTable 8. Cox proportional hazards regression analysis
eTable 9. Analysis excluding children with cerebral palsy, intellectual disability, and epilepsy
eTable 10. The association between combinations of Apgar score and umbilical cord blood pH and ADHD in children born 2004-2011 and children born 2012-2018 using complete data
eTable 11. The association between combinations of Apgar score and umbilical cord blood pH and ADHD in children born 2004-2011 and children born 2012-2018 using imputed data
eTable 12. Distribution of observed and imputed Apgar score
eTable 13. Distribution of observed and imputed umbilical cord pH according to Apgar score category
eReferences

This supplemental material has been provided by the authors to give readers additional information about their work.

eFigure: Directed Acyclic Graph on the association between perinatal hypoxia and ADHD

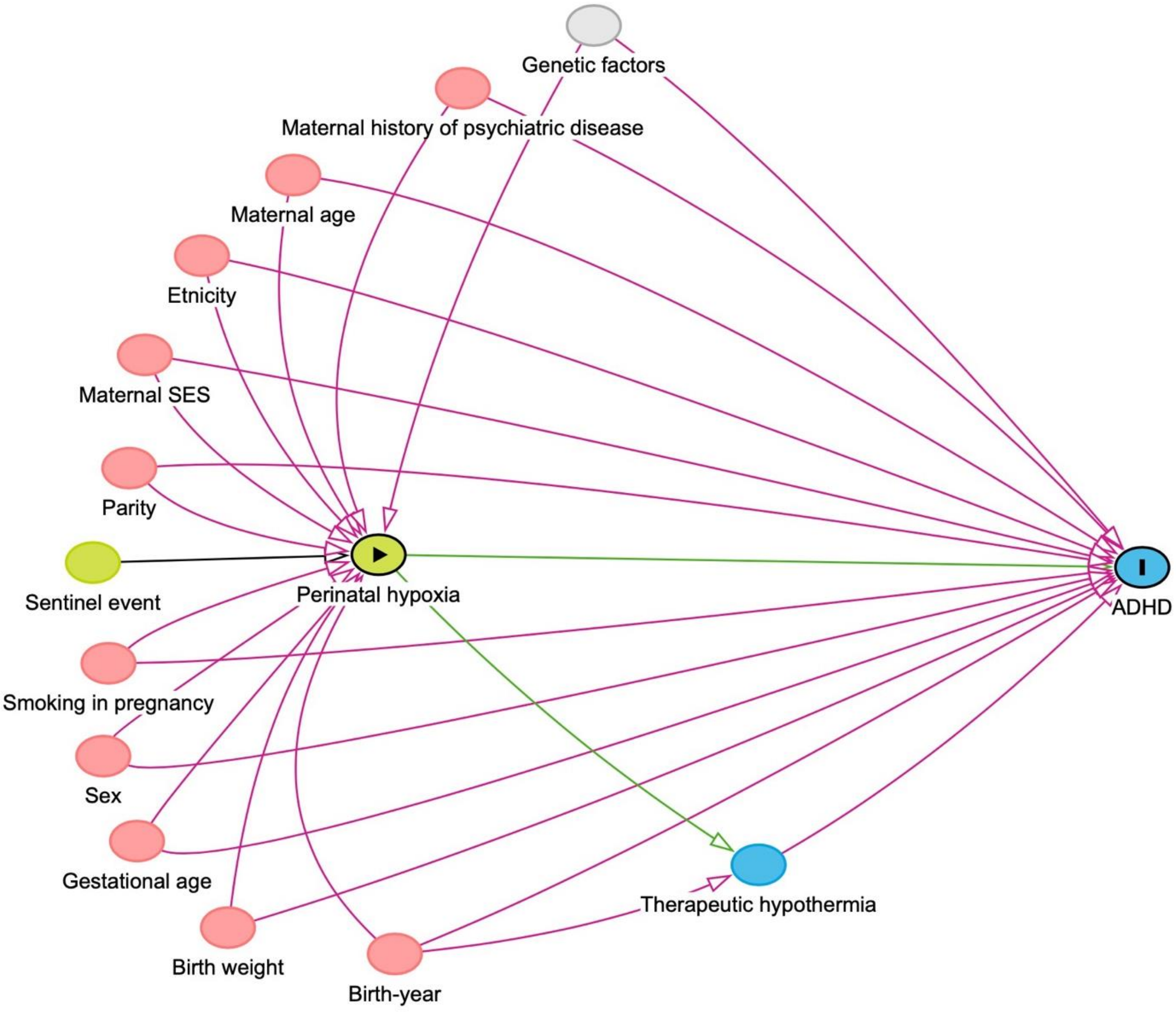

Red: confounders adjusted for, Grey: unobserved, Green: on the causal path not adjusted for.

eTable 1. Data sources

Data source	Description	Variables obtained from the registry
The Central Person Registry[1]	Holds information on all Danish citizens which includes citizenship, date of death, unknown whereabouts, and emigration. Registration is required by law.	Death date<br>Emigration date<br>Ethnicity
Medical Birth Registry[2]	Holds information on all births in Denmark. The registry covers information on both the pregnancy, the pregnant woman, and the offspring.	Birth date<br>Apgar score<br>Umbilical cord blood pH<br>Gestational age<br>Birth weight<br>Sex<br>Parity<br>Maternal age<br>Smoking in pregnancy
The Danish National Patient Registry[3]	Holds information on all hospital contacts including admissions and outpatient visits. For each contact the registry contains information on primary and secondary diagnoses (according to ICD-10), date of admission and date of discharge.	Child ADHD diagnosis<br>Maternal psychiatric diagnoses
The Danish Psychiatric Central Registry[4]	Holds information on all psychiatric admissions prior to 1995. For each contact primary and secondary diagnoses, admission date and date of discharge are registered.	Maternal psychiatric diagnoses
The Danish National Prescription Registry[5]	Holds information on prescriptions dispensed at all Danish Pharmacies. Information include ATC-codes and dispensing date.	Prescriptions for ADHD treatment of the child.
Statistics Denmark[6]	Statistics Denmark manage several registries which holds information on socioeconomics.	Maternal education<br>Family income

eMethods. Multiple imputation model

We used multiple imputation by chained equations to impute missing values for umbilical cord pH, Apgar score, gestational age, birth weight, maternal smoking status in pregnancy, and maternal education category. For imputation of continuous variables (umbilical cord pH, gestational age, birth weight) we used linear regression as imputation method. Non-linearity was handled with log-transformation. Missing values of Apgar score and maternal education categories were imputed with ordinal logistic regression. Maternal smoking status in pregnancy was imputed as categorical with logistic regression. For continuous variables as predictors, we used fractional polynomials according to the Royston and Altman model selection algorithm to identify the best prediction model for each imputed variable.[7] All variables in the adjusted model (including the outcome) were included in the multiple imputation model. Auxiliary variables were included in the multiple imputation model if they were associated with the missingness *and* the non-missing values of any of the imputed variables. Child head circumference at birth, child length at birth, gestational age, and birthweight were used as continuous predictors and auxiliary variables in the imputation model. The following variables, identified through diagnosis codes from the International Classification of Diseases 10th revision (ICD-10) and procedure codes from the Danish Helath Care Classification System (SKS) were used as auxiliary categorical predictors: scalp blood sampling for pH or lactate (taken or not taken, SKS: BMBA03, ZZ4224, ZZ4227), labor and birth complicated by fetal distress (ICD-10 DO68*), abruptio placentae (ICD-10: DO45), operative vaginal delivery (SKS: KMAE*), umbilical cord prolapse (ICD-10: DO690), uterus rupture (ICD-10: DO711*, DO758A, SKS: KMCC00), acute caesarean section (SKS: KMCA10A, KMCA10E, KMCA12B), elective caesarean section (SKS: KMCA10B, KMCA10D), pharmacological analgesia during labor (SKS: BABZ00, BAFA7, BAHY0, NAAD0B, NAAD52C), pharmacological stimulation of contractions (SKS: BKHD3*), induction of labor (SKS BKHD2*, KMAC00, KMAC96A), birth at a highly specialized hospital, neonatal diagnosis of asphyxia (ICD-10: DP210), neonatal treatment with CPAP (SKS: BGFC32), neonatal treatment with mechanical ventilator (SKS: BGDA0*), neonatal intensive care admission, neonatal sepsis (ICD-10: DP36*), child intellectual disability diagnosis (ICD-10: DF7*, DR620), polyhydramnios (ICD-10: DO40*) , preeclampsia (ICD-10: DO11, DO13*, DO14*, DO15*, DO16*), and gestational diabetes (ICD10: DO244*).
We created 30 imputed datasets with 10 burn-in iterations. Convergence was assessed by visual inspection of plots of summaries of mean and standard deviation against iteration numbers. The fit of the imputation model was evaluated by plotting distributions and proportions of imputed, observed, and total (imputed and observed data combined) data.[8]

eAppendix. Sibling analysis

For the sibling analysis we defined two exposure groups: 1) A hypoxia group with pH <7.20 and Apgar 0-6 and 2) A reference group with pH ≥7.20 and Apgar of 7-10. Discordant sibling pairs were compared by use of conditional fixed effects model.
We identified 56 discordant pairs, we estimated adjusted OR 1.62 (95%CI, 0.87-3.00) for ADHD for those with Apgar 0-6 combined with pH less than 7.20 compared to the reference category.

eTable 2. The association between Apgar score alone or umbilical cord blood pH alone and ADHD.

Odds ratios for ADHD according to Apgar score alone or umbilical cord blood pH alone.

	**Crude OR (95%CI)[a]**	**Adjusted OR (95%CI)[b]**
	n=819,658	n=819,658
**Apgar category:**		
7-10	Ref	Ref
4-6	1.20 (1.00;1.43)	1.07 (0.89;1.28)
0-3	1.44 (1.08;1.94)	1.32 (0.98;1.80)
**pH category:**		
$\geq$ 7.20	Ref	Ref
7.10-7.19	1.04 (1.00;1.08)	1.02 (0.97;1.06)
<7.10	1.10 (1.01;1.19)	1.09 (1.00;1.18)

[a]Crude model: Logistic regression using imputed data adjusted for birth year.

[b]Adjusted model: Logistic regression using imputed data. Odds ratio adjusted for birth year, sex, gestational age, birth weight, maternal age, parity, smoking in pregnancy, maternal history of psychiatric disease, maternal education, family income, child ethnicity.

eTable 3. The association between umbilical cord blood pH alone and ADHD. Including a separate category for pH<7.00

Odds ratios for umbilical cord blood pH alone and ADHD.

	**Crude OR (95%CI)[a]**	**Adjusted OR (95%CI)[b]**
pH category		
≥7.20	Ref	Ref
7.10-7.19	1.04 (1.00;1.08)	1.01 (0.97;1.06)
7.00-7.10	1.10 (1.01;1.20)	1.09 (1.00;1.19)
<7.00	1.08 (0.90;1.29)	1.07 (0.89;1.28)

[a]Crude model: Logistic regression using imputed data adjusted for birth year.

[b]Adjusted model: Logistic regression using imputed data. Odds ratio adjusted for birth year, sex, gestational age, birth weight, maternal age, parity, smoking in pregnancy, maternal history of psychiatric disease, maternal education, family income, child ethnicity.

eTable 4. The association between combinations of Apgar score and umbilical cord blood pH and ADHD using imputed data.

Odds ratios for ADHD stratified by umbilical cord blood pH *and* Apgar score

		**Crude OR (95%CI)[a]**	**Adjusted OR (95%CI)[b]**
		n=819,658	n=819,658
**pH category**	**Apgar category**		
≥7.20	7-10	Ref	Ref
≥7.20	4-6	1.11 (0.84;1.46)	0.95 (0.71;1.26)
≥7.20	0-3	1.16 (0.72;1.84)	1.05 (0.65;1.69)
7.10-7.19	7-10	1.03 (0.99;1.08)	1.02 (0.97;1.06)
7.10-7.19	4-6	1.36 (0.96;1.92)	1.26 (0.91;1.76)
7.10-7.19	0-3	1.62 (0.88;3.00)	1.42 (0.75;2.67)
<7.10	7-10	1.08 (1.00;1.18)	1.08 (0.99;1.17)
<7.10	4-6	1.22 (0.86;1.72)	1.11 (0.78;1.58)
<7.10	0-3	1.92 (1.10;3.34)	1.86 (1.04;3.33)

[a]Crude model: Logistic regression using imputed data, adjusted for birth year.

[b]Adjusted model: Logistic regression using imputed data, adjusted for birth year, sex, gestational age, birth weight, maternal age, parity, smoking in pregnancy, maternal history of psychiatric disease, maternal education, family income, child ethnicity.

eTable 5. Complete data analysis

Odds ratios for ADHD stratified by umbilical cord blood pH *and* Apgar score.

		Crude OR (95% CI)[a]	Adjusted OR (95% CI)[b]
		n=637,004	n=617,966
**pH category**	**Apgar category**		
≥7.20	7-10	Ref	Ref
≥7.20	4-6	1.06 (0.77;1.47)	0.81 (0.57;1.15)
≥7.20	0-3	1.18 (0.67;2.07)	1.11 (0.63;1.98)
7.10-7.19	7-10	1.02 (0.98;1.07)	1.01 (0.97;1.05)
7.10-7.19	4-6	1.53 (1.06;2.22)	1.42 (0.97;2.08)
7.10-7.19	0-3	1.75 (0.85;3.59)	1.62 (0.78;3.39)
<7.10	7-10	1.08 (0.99;1.16)	1.07 (0.99;1.17)
<7.10	4-6	1.24 (0.86;1.79)	1.16 (0.80;1.69)
<7.10	0-3	1.92 (1.06;3.49)	1.90 (0.99;3.64)

[a]Crude model: Logistic regression using complete data adjusted for birth year.

[b]Adjusted model: Logistic regression using complete data adjusted for birth year, sex, gestational age, maternal age, parity, smoking in pregnancy, maternal history of psychiatric disease, maternal education, family income, child ethnicity.

eTable 6. Analysis with exposure categorized according to validated arterial values

Odds ratios of ADHD stratified by umbilical cord blood pH *and* Apgar score. Exposure category defined by validated arterial values. Arterial pH validated by at least two pH registrations with difference ≥0.02.

		**Crude OR (95% CI)[a]**	**Adjusted OR (95% CI)[b]**
		n=335,372	n=326,797
**pH category**	**Apgar category**		
≥7.20	7-10	Ref	Ref
≥7.20	4-6	1.15 (0.63;2.10)	0.96 (0.53:1;75)
≥7.20	0-3	1.57 (0.68;3.58)	1.21 (0.51;2.86)
7.10-7.19	7-10	1.00 (0.93;1.06)	0.96 (0.90;1.03)
7.10-7.19	4-6	1.25 (0.64;2.45)	1.22 (0.62;2.39)
7.10-7.19	0-3	1.31 (0.32;5.46)	1.32 (0.32;5.50)
<7.10	7-10	1.06 (0.92;1.22)	1.04 (0.91;1.20)
<7.10	4-6	1.26 (0.67;2.36)	1.17 (0.62;2.21)
<7.10	0-3	1.87 (0.67;5.29)	1.97 (0.70;5.57)

[a]Crude model: Logistic regression using complete data adjusted for birth year.
[b]Adjusted model: Logistic regression using complete data adjusted for birth year, sex, gestational age, maternal age, parity, smoking in pregnancy, maternal history of psychiatric disease, maternal education, family income, child ethnicity.

eTable 7. Logbinomial regression analysis

Relative risk ratio of ADHD stratified by umbilical cord blood pH *and* Apgar score.

		**Crude RR (95% CI)[a]**	**Adjusted RR (95% CI)[b]**
		n=819,658	n=819,658
**pH category**	**Apgar category**		
≥7.20	7-10	Ref	Ref
≥7.20	4-6	1.10 (0.85;1.44)	0.95 (0.73;1.23)
≥7.20	0-3	1.14 (0.73;1.76)	1.02 (0.66;1.59)
7.10-7.19	7-10	1.03 (0.99;1.08)	1.01 (0.97;1.06)
7.10-7.19	4-6	1.35 (0.99;1.83)	1.24 (0.91;1.68)
7.10-7.19	0-3	1.57 (0.89;2.78)	1.34 (0.76;2.36)
<7.10	7-10	1.08 (1.00 (1.17)	1.07 (0.99;1.16)
<7.10	4-6	1.21 (0.877;1.67)	1.10 (0.80;1.52)
<7.10	0-3	1.83 (1.09 (3.05)	1.69 (1.01;2.85)

[a]Crude model: Log-binomial regression using imputed data adjusted for birth year.

[b]Adjusted model: Log-binomial regression using imputed data adjusted for birth year, sex, gestational age, maternal age, parity, smoking in pregnancy, maternal history of psychiatric disease, maternal education, family income, child ethnicity.

eTable 8. Cox proportional hazards regression analysis

Hazard ratio of ADHD stratified by umbilical cord blood pH *and* Apgar score.
Censoring deaths and emigration from birth until Dec 31, 2022.

		**Crude HR (95% CI)[a]**	**Adjusted HR (95% CI)[b]**
		n=819,658	n=819,658
**pH category**	**Apgar category**		
≥7.20	7-10	Ref	Ref
≥7.20	4-6	1.12 (0.85;1.47)	0.96 (0.73;1.26)
≥7.20	0-3	1.15 (0.73;1.82)	1.04 (0.66;1.64)
7.10-7.19	7-10	1.03 (0.99;1.08)	1.02 (0.97;1.06)
7.10-7.19	4-6	1.35 (0.97;1.86)	1.23 (0.89;1.71)
7.10-7.19	0-3	1.57 (0.86:2.86)	1.37 (0.75;2.50)
<7.10	7-10	1.08 (1.00;1.17)	1.07 (0.99;1.16)
<7.10	4-6	1.22 (0.87;1.70)	1.12 (0.80;1.57)
<7.10	0-3	1.58 (0.88;2.83)	1.49 (0.83;2.69)

[a]Crude model: Cox proportional hazards regression using imputed data adjusted for birth year. [b]Adjusted model: Cox proportional hazards regression using imputed data adjusted for birth year, sex, gestational age, maternal age, parity, smoking in pregnancy, maternal history of psychiatric disease, maternal education, family income, child ethnicity.

eTable 9. Analysis excluding children with cerebral palsy, intellectual disability, and epilepsy

Odds ratios of ADHD stratified by umbilical cord blood pH *and* Apgar score.
Cerebral palsy defined as registration with any diagnosis of cerebral palsy in the Danish Cerebral Palsy Follow up program.[9] Intellectual disability defined as any contact with a diagnosis of DF7* or DR620* in the Danish National Patient Registry or the Danish Psychiatric Central Registry. Epilepsy defined as any contact with DG40* (DG403P and DG404C not included) in the Danish National Patient Registry or any prescription with medicine with ATC-codes N03A* or N05BA09 in the Danish National Prescription Registry.

		**Crude OR (95%CI)**[a]	**Adjusted OR (95%CI)**[b]
		n=819,658	n=819,658
**pH category**	**Apgar category**		
≥7.20	7-10	Ref	Ref
≥7.20	4-6	1.12 (0.83;1.50)	0.97 (0.72;1.31)
≥7.20	0-3	1.14 (0.69;1.88)	1.04 (0.63;1.73)
7.10-7.19	7-10	1.04 (0.99;1.09)	1.02 (0.98;1.07)
7.10-7.19	4-6	1.31 (0.92;1.87)	1.22 (0.85;1.74)
7.10-7.19	0-3	1.65 (0.87;3.11)	1.45 (0.76;2.80)
<7.10	7-10	1.09 (1.00;1.19)	1.08 (0.99;1.18)
<7.10	4-6	1.28 (0.89;1.84)	1.19 (0.82;1.72)
<7.10	0-3	1.72 (0.88;3.36)	1.68 (0.85;3.35)

[a]Crude model: Logistic regression using imputed data, adjusted for birth year.
[b]Adjusted model: Logistic regression using imputed data, adjusted for birth year, sex, gestational age, birth weight, maternal age, parity, smoking in pregnancy, maternal history of psychiatric disease, maternal education, family income, child ethnicity.

eTable 10. The association between combinations of Apgar score and umbilical cord blood pH and ADHD in children born 2004-2011 and children born 2012-2018 using complete data.

Odds ratios of ADHD stratified by umbilical cord blood pH *and* Apgar score in children born 2004-2018, children born 2004-2011 and children born 2012-2018.

**Complete data**		**Total cohort Children born 2004-2018**		**Children born 2004-2011**		**Children born 2004-2011 (restricted follow-up)**		**Children born 2012-2018**	
		**Crude OR (95%CI)[a]**	**Adjusted OR (95%CI)[b]**	**Crude OR (95%CI)[a]**	**Adjusted OR (95%CI)[b]**	**Crude OR (95%CI)[a,c]**	**Adjusted OR (95%CI)[b,c]**	**Crude OR (95%CI)[a]**	**Adjusted OR (95%CI)[b]**
		n=637,004	n=617,966	n= 295,948	n= 286,080	n= 295,948	n= 286,080	n=341,056	n=331,886
**pH category**	**Apgar category**								
≥7.20	7-10	Ref	Ref	Ref	Ref	Ref	Ref	Ref	Ref
≥7.20	0-6	1.09 (0.82;1.44)	0.88 (0.65;1.19)	0.96 (0.69;1.35)	0.78 (0.54;1.12)	1.35 (0.72;2.52)	1.08 (0.56;2.08)	1.51 (0.92;2.49)	1.16 (0.69;1.96)
<7.20	7-10	1.03 (0.99;1.07)	1.02 (0.98;1.06)	1.03 (0.99;1.08)	1.02 (0.98;1.07)	1.05 (0.95;1.15)	1.02 (0.93;1.12)	1.03 (0.96;1.11)	1.00 (0.93;1.08)
<7.20	0-6	1.47 (1.17;1.84)	1.37 (1.09;1.73)	1.44 (1.11;1.87)	1.34 (1.02;1.76)	1.49 (0.88;2.54)	1.28 (0.73;2.24)	1.54 (0.97;2.43)	1.46 (0.93;2.30)

[a]Crude model: Logistic regression using complete data, adjusted for birth year.

[b]Adjusted model: Logistic regression using complete data, adjusted for birth year, sex, gestational age, maternal age, parity, smoking in pregnancy, maternal history of psychiatric disease, maternal education, family income, child ethnicity.

[c]Follow-up restricted until Dec 31, 2015, making follow-up time comparable for the two birth cohorts.

eTable 11. The association between combinations of Apgar score and umbilical cord blood pH and ADHD in children born 2004-2011 and children born 2012-2018 using imputed data.

Odds ratios of ADHD stratified by umbilical cord blood pH *and* Apgar score in children born 2004-2018, children born 2004-2011 and children born 2012-2018.

Imputed data		Total cohort Children born 2004-2018		Children born 2004-2011		Children born 2004-2011 (restricted follow-up)		Children born 2012-2018	
		**Crude OR (95%CI)[a]**	**Adjusted OR (95%CI)[b]**	**Crude OR (95%CI)[a]**	**Adjusted OR (95%CI)[b]**	**Crude OR (95%CI)[a,c]**	**Adjusted OR (95%CI)[b,c]**	**Crude OR (95%CI)[a]**	**Adjusted OR (95%CI)[b]**
		n=819,658	n=819,658	n=450,844	n=450,844	n=450,844	n=450,844	n=368,814	n=368,814
**pH category**	**Apgar category**								
≥7.20	7-10	Ref	Ref	Ref	Ref	Ref	Ref	Ref	Ref
≥7.20	0-6	1.12 (0.88;1.42)	0.97 (0.76;1.24)	1.00 (0.76;1.32)	0.88 (0.66;1.16)	1.41 (0.87;2.28)	1.20 (0.73;1.95)	1.70 (1.07;2.71)	1.37 (0.85;2.21)
<7.20	7-10	1.04 (1.00;1.08)	1.02 (0.98;1.07)	1.04 (1.00;1.09)	1.03 (0.98;1.08)	1.06 (0.97;1.16)	1.03 (0.95;1.13)	1.03 (0.96;1.11)	1.00 (0.93;1.07)
<7.20	0-6	1.40 (1.14;1.71)	1.29 (1.01;1.58)	1.37 (1.09;1.72)	1.26 (1.00;1.60)	1.52 (0.97;2.38)	1.33 (0.85;2.10)	1.51 (0.97;2.36)	1.38 (0.89;2.15)

[a]Crude model: Logistic regression using imputed data, adjusted for birth year.

[b]Adjusted model: Logistic regression using imputed data, adjusted for birth year, sex, gestational age, birth weight, maternal age, parity, smoking in pregnancy, maternal history of psychiatric disease, maternal education, family income, child ethnicity.

[c]Follow-up restricted until Dec 31, 2015, making follow-up time comparable for the two birth cohorts.

eTable 12. Distribution of observed and imputed Apgar score.

Distribution of observed Apgar score in the total population with available Apgar score, of imputed Apgar scores in the total population with missing Apgar score, of observed Apgar score in a random sample of 1% of the population, and of imputed Apgar scores in the random 1% sample.

	Total population		Random sample of 1%	
**Apgar score category**	**Distribution of observed Apgar scores No. (%)**	**Distribution of imputed Apgar scores No. (%)**	**Distribution of observed Apgar scores No. (%)**	**Distribution of imputed Apgar scores No. (%)**
**Total**	814,192	5020	8197	8197
**7-10**	810,192 (99.45%)	~ 4,929 (98.20%)	8142 (99.33 %)	~8148 (99.40 %)
**4-6**	3,390 (0.42%)	~ 65 (1.29%)	41 (0.50 %)	~38 (0.46 %)
**0-3**	1,056 (0.13%)	~26 (0.52%)	14 (0.17 %)	~11 (0.14 %)

eTable 13. Distribution of observed and imputed umbilical cord pH according to Apgar score category

a) Observed and imputed distribution of pH according to Apgar score in the total population

	pH <7.10<br>No. (%)	pH 7.10-7.19<br>No. (%)	pH ≥ 7.20<br>No. (%)
**Total**			
Observed	22,813 (4%)	116,790 (18%)	499,148 (78%)
Imputed	~ 3163 (2%)	~ 27,096 (15%)	~150,648 (83%)
**Apgar score 0-3**			
Observed	249 (30%)	187 (23%)	383 (47%)
Imputed	~ 44 (17%)	~ 72 (29%)	~ 130 (54%)
**Apgar score 4-6**			
Observed	841 (30%)	724 (26%)	1,199 (43%)
Imputed	~ 107 (16%)	~ 213 (33%)	~ 325 (51%)
**Apgar score 7-10**			
Observed	21,521 (3%)	115,505 (18%)	496,395 (78%)
Imputed	~ 3,012 (2%)	~ 26,812 (15%)	~ 150,193 (84%)

b) Observed and imputed distribution of pH according to Apgar score in a random sample of 22% with observed pH

	pH <7.10<br>No. (%)	pH 7.10-7.19<br>No. (%)	pH ≥ 7.20<br>No. (%)
**Total**			
Observed	4,886 (4%)	25,579 (18%)	109,676 (78%)
Imputed	~ 5,025 (4%)	~ 29,853 (21%)	~ 109,935 (76%)
**Apgar score 0-3**			
Observed	53 (29%)	50 (28%)	77 (42%)
Imputed	~ 51 (27%)	~ 64 (34%)	~ 71 (38%)
**Apgar score 4-6**			
Observed	178 (29%)	159 (26%)	271 (45%)
Imputed	~ 132 (21%)	~ 210 (34%)	~ 286 (46%)
**Apgar score 7-10**			
Observed	4,655 (3%)	25,370 (18%)	109,328 (78%)
Imputed	~ 4842 (3%)	~ 29,579 (21%)	~ 109, 578 (76%)

Note: Percentages were calculated within each Apgar score category (row percentages)

eReferences

1 Schmidt, M., Pedersen, L. & Sørensen, H. T. The Danish Civil Registration System as a Tool in Epidemiology. *Eur J Epidemiol* **29**, 541-549 (2014).

2 Bliddal, M., Broe, A., Pottegård, A., Olsen, J. & Langhoff-Roos, J. The Danish Medical Birth Register. *Eur J Epidemiol* **33**, 27-36 (2018).

3 Schmidt, M. et al. The Danish National Patient Registry: A Review of Content, Data Quality, and Research Potential. *Clin Epidemiol* **7**, 449-490 (2015).

4 Mors, O., Perto, G. P. & Mortensen, P. B. The Danish Psychiatric Central Research Register. *Scand J Public Health* **39**, 54-57 (2011).

5 Kildemoes, H. W., Sørensen, H. T. & Hallas, J. The Danish National Prescription Registry. *Scand J Public Health* **39**, 38-41 (2011).

6 *Statistics Denmark*, <https://www.dst.dk/en. Assessed June 19, 2015> (

7 White, I. R., Royston, P. & Wood, A. M. Multiple Imputation Using Chained Equations: Issues and Guidance for Practice. *Stat Med* **30**, 377-399 (2011).

8 Eddings, W. & Marchenko, Y. Diagnostics for Multiple Imputation in Stata. *The Stata Journal* **12**, 353-367 (2012).

9 Larsen, M. L., Hoei-Hansen, C. E. & Rackauskaite, G. The Diagnosis of Cerebral Palsy in Two Danish National Registries: A Validation Study. *Scand J Public Health* **53**, 1-7 (2025).
