# Supplementary material for: Umbilical Cord Blood pH Level, Apgar Score, and Attention-Deficit/Hyperactivity Disorder
Source: JAMA Netw Open. 2026 Jan 26;9(1):e2554672. doi: 10.1001/jamanetworkopen.2025.54672 (PMC12836134; doi:10.1001/jamanetworkopen.2025.54672)
Supplement: Supplement 2. — Data Sharing Statement [file jamanetwopen-e2554672-s002.pdf]

Data Sharing Statement

Pedersen. Umbilical Cord Blood pH Level, Apgar Score, and Attention-Deficit/Hyperactivity Disorder. *JAMA Netw Open*. Published January 26, 2026.
doi:10.1001/jamanetworkopen.2025.54672

Data
Data available: No

Additional Information
Explanation for why data not available: The data is not available from the corresponding author to others according to Danish data protection legislation. The data can be obtained from Danish registries upon application.
